# Comparison of Femtosecond Laser-Assisted and Ultrasound-Assisted Cataract Surgery with Focus on Endothelial Analysis

**DOI:** 10.3390/s21030996

**Published:** 2021-02-02

**Authors:** Anna Schroeter, Martina Kropp, Zeljka Cvejic, Gabriele Thumann, Bojan Pajic

**Affiliations:** 1 Department of Ophthalmology, Basel University Hospital, Mittlerestr str. 91, 4056 Basel, Switzerland; anna.schroeter@gmail.com; 2 Division of Ophthalmology, Department of Clinical Neurosciences, Geneva University Hospitals, 1205 Geneva, Switzerland; martina.kropp@unige.ch (M.K.); gabriele.thumann@hcuge.ch (G.T.); 3 Faculty of Medicine, University of Geneva, 1205 Geneva, Switzerland; 4 Department of Physics, Faculty of Sciences, University of Novi Sad, Trg Dositeja Obradovica 4, 21000 Novi Sad, Serbia; zeljka.cvejic@df.uns.ac.rs; 5 Eye Clinic Orasis, Swiss Eye Research Foundation, 5734 Reinach, Switzerland; 6 Faculty of Medicine of the Military Medical Academy, University of Defence, 11000 Belgrade, Serbia

**Keywords:** femtosecond laser, cataract surgery, endothelial cell analysis

## Abstract

Femtosecond laser-assisted cataract surgery has the potential to make critical steps of cataract surgery easier and safer, and reduce endothelial cell loss, thus, improving postoperative outcomes. This study compared FLACS with the conventional method in terms of endothelial cells behavior, clinical outcomes, and capsulotomy precision. Methods: In a single-center, randomized controlled study, 130 patients with cataracta senilis received FLACS or conventional cataract surgery. Results: A significant endothelial cell loss was observed postoperatively, compared to the preoperative values in both groups. The endothelial cell counts was significantly better in the FLACS group in cataract grade 2 (*p* = 0.048) patients, compared to conventionally at 4 weeks. The effective phaco time was notably shorter in grade 2 of the FLACS group (*p* = 0.007) compared to the conventional. However, no statistically significant differences were found for the whole sample, including all cataract grades, due to the overall cataract density in the FLACS group being significantly higher (2.60 ± 0.58, *p* < 0.001) as compared to conventional methods (2.23 ± 0.42). Conclusions: Low energy FLACS provides a better result compared to endothelial cell loss, size, and shape variations, as well as in effective phaco time within certain cataract grade subgroups. A complete comparison between two groups was not possible because of the higher cataract grade in the FLACS. FLACS displayed a positive effect on endothelial cell preservation and was proven to be much more precise.

## 1. Introduction

The assessment of endothelial cell density is very important before any intraocular surgery. Intraocular surgery can cause endothelial cell loss, which in extreme cases can lead to corneal decompensation [1]. Factors that can lead to endothelial cell loss include intraoperative destructive toxic and mechanical variables, such as turbulent fluid flows, turbulent lens fragments, ultrasonic energy from conventional ultrasound cataract surgery, contact with surgical instruments, intraocular lens implantation, and pharmacological influences.

**Figure 1:**
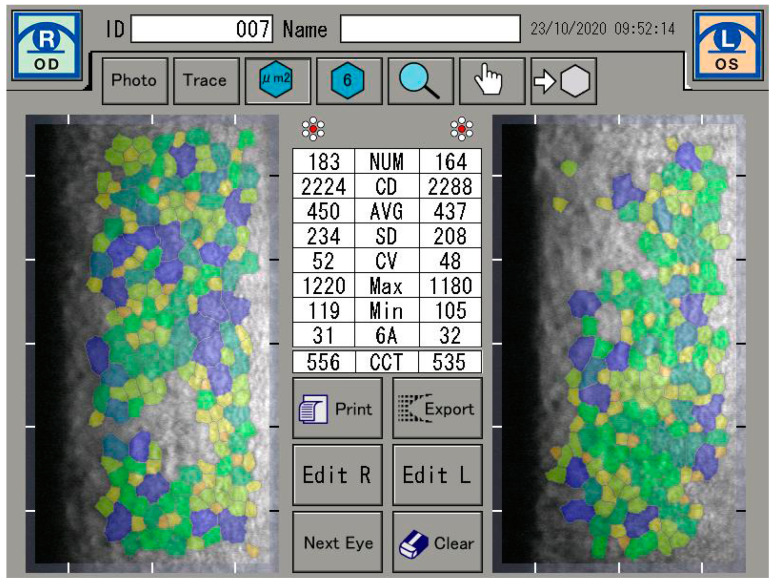
Endothelial cell analysis.

If a defect in endothelial cells occurs, endothelial cell continuity can be ensured by the migration and expansion of neighboring cells. It is also known that with age, the proportion of hexagonal cells decreases (pleomorphism) and the coefficient of variation of the cell area increases [1,2]. However, the reduction in endothelial cell count is not only caused by age, but can often be seen as a result of cataract surgery, long-term contact lens wear, diabetes mellitus, inflammation, and keratoconus [3,4,5,6]. In this sense, the functional state of the cornea is described by the coefficient of variation of the endothelial cell area (CV) and the percentage of hexagonal endothelial cells (6A). A healthy cornea consists of at least 60% hexagonal endothelial cells and a coefficient of variation of less than 40 [7] (Figure 1).

The corneal endothelium has an extraordinary functional reserve. There is no precise correlation between corneal function and cell density. However, there is a high probability that corneal decompensation might occur if the cell density falls below 500 cells/mm^2^ (range 750–250 cells/mm^2^) [1,8,9].

The infrared beam of light of a femto second laser precisely separates tissue through a process called photo-disruption. The laser emits optical pulses with a duration as short as one-quadrillionth of a second ($10^{-15}$ = femtosecond). Due to the short pulse duration, energy in the nano-joule range is applied to achieve an optical breakdown. With this low energy and short pulse duration combination, a negligible amount of heat is transferred in the tissue, hardly causing any collateral tissue damage [10,11].

The femtosecond laser proved to be an effective and precise means for the creation of clear corneal incisions, lens fragmentation, and arc incisions for the correction of residual astigmatism during cataract surgery. In addition, the centration, size, and circularity of the capsulotomy created by the femtosecond laser play a pivotal role in terms of effective lens position and postoperative refractive results. Since the ultrasound source of the phacoemulsification procedure distributes relatively high-energy that can cause damage to healthy cells during cataract surgery, the implementation of the femtosecond laser could extensively reduce or even eliminate the use of phaco energy, contributing to a reduction in the amount of postsurgical inflammation and trauma of intraocular tissues, e.g., corneal endothelium and capsule, ultimately enhancing the visual outcomes [11,12].

Phacoemulsification, an ultrasound procedure, was introduced into cataract surgery just over 40 years ago and has become the standard method for more than 20 years [13,14]. Nevertheless, the ultrasound source of phacoemulsification distributes relatively high-energy and can cause damage to healthy cells during cataract surgery, especially to the corneal endothelium [15]. A further improvement in the cataract surgery using femtosecond lasers can potentially facilitate eye surgery, but above all it can be more precise [16].

Hence, the aim of the study was to analyze the clinical outcome with a specific focus on corneal endothelial cell behavior, when femtosecond laser and conventional cataract surgery were compared.

## 2. Materials and Methods

The randomized, single-center controlled study was conducted at the ORASIS eye clinic in Switzerland. Two different surgical methods were used for cataract surgery and compared with each other—femtosecond laser assisted cataract surgery (FLACS) and conventional ultrasound cataract surgery (CUCS). The corneal analysis and clinical outcome were the primary objectives of the research. One hundred and thirty patients’ 130 eyes were included in the study. All surgeries were performed by the same experienced surgeons (B.P.). Sixty-five eyes were randomly assigned to either receive FLACS and 65 eyes to receive CUCS. Informed consent was obtained after the purpose and aim of the study were explained in detail to all. Out of 130 patients, 126 patients completed the study. Four patients in total, with two from the FLACS and two from the CUCS group, were excluded from the study, as they did not show up for all postoperative examinations. The Study protocol (2020-01242) was approved by the Ethics Committee Nordwest-und Zentralschweiz (EKNZ, Switzerland). Table 1 lists all expressions and abbreviations.

### 2.1. Inclusion/Exclusion Studies Criteria

The inclusion criteria required the presence of a clear cornea and the patient’s age being 50 years or more. Another important point was the feasibility of attaching a docking system of the femtosecond laser with cataract extraction and fitting of a primary intraocular lens. The patient was to be willing and able to attend the scheduled follow-up examinations.

The exclusion criteria of the study were as follows—patients were excluded from the study when the minimum and maximum K-values of the central 3-mm corneal zone differed with more than 5 D, meaning that the steepest meridian should not exceed 48 D, and in particular, it should not be flatter than 37 D in the other meridian; all corneal diseases, such as corneal scarring, which could impede the transmission of the laser wavelength or distort the laser light were excluded; poorly dilated pupils or other pupillary defects were not considered for the study.

In addition, other ocular diseases that led to the exclusion were manifest glaucoma, ocular hypertension, pseudoexfoliation, and any kind of former corneal or lens surgery. Lens instability with respect to lentodonesis and keratoconic changes were not included. Furthermore, not all metabolic and autoimmune diseases were considered for the study. The anterior chamber depth (ACD) should not be less than 1.5 and greater than 4.8 mm. Patients who participated in other studies at the same time were also excluded.

### 2.2. Specular Microscopy of the Corneal Endothelium

The specular microscope is a reflected light, microscope. When incident light comes to an anterior corneal surface, it can be reflected, as well as absorbed or refracted, i.e., transmitted further through corneal tissue. Transmitted light that reaches the posterior corneal surface is mostly transmitted into aqueous humour, and only 0.22% is reflected due to change in index of refraction at the endothelium-aqueous humor interface. The specular reflex occurs at regular, ideally flat-surfaced interface of two refractive indices, whereby the light ray from the object has an angle of incidence equal to the angle of reflection for the observer. The light reflected specularly from the posterior corneal surface is collected through a focused system [9,17]. The corneal endothelium was analyzed by a specular microscopy (EM-3000). The EM-3000 uses an optical magnification of ×190, and up to 300 cells per image are counted in an area of 0.1 mm^2^ (recording an area of 0.25 mm × 0.54 mm) [18,19]. The unit captured 15 images per measurement and the images were automatically sorted and displayed by the software, according to image quality, from best to worst condition. The best image among the capture results was evaluated. Preoperative, 4-week and 8-week postoperative measurements were taken. The analysis were focused on the absolute number of endothelial cells (NUM), the density of endothelial cells per mm^2^ (CD), and on the average size of the endothelial cells. Furthermore, the coefficient of variation derived by dividing the standard deviation of the average size (CV) was investigated, as well as the range of the largest (Max) and the smallest cell (Min) and the percentage of hexagonal endothelial cells. The central corneal thickness (CCT) was also measured (Figure 1).

### 2.3. Optical Coherence Tomography Imaging

Optical coherence tomography (OCT) is one of the most important imaging methods in ophthalmology. This high-resolution, non-invasive optical imaging technique for transparent or scattering media is used simultaneously with the femtosecond laser. The working principle of OCT is very similar to the working principle of medical ultrasonic devices. This imaging technique directs waves to the tissue where the waves are reflected as echo. The magnitude of back-reflected waves are measured and analyzed, and compared to the reference one. Generally, waves delay is measured to dissolve the depth at which reflection occurred. In OCT, the near-infrared light is used, which travels much faster than ultrasound. The low-coherence interferometry is the basic principle for all OCT applications.

Fourier domain OCT imaging is applied in the FEMTO LDV Z8 as the spectral domain OCT (SD-OCT). The heart of any OCT system is the light interference setup and is essentially based on the Michelson light interferometer setup. A small interference packet is measured for each boundary layer along the axis. From the positions of the scanner mirror, the interference effects are measured with the optical path length [20,21]. In the Fourier domain (FD), the interferometer is not mechanically modified. The simple intensity detection is replaced by a spectrally resolved detection. This means that in FD-OCT, the light simultaneously echoes come from all axial depths. All light echoes are detected as modulations in the source spectrum and recorded simultaneously with all other spectral components. The FD-OCT system has a static mirror This feature of FD-OCT eliminates the moving mirror and the limitations imposed by the mechanical translation and inertia of this mechanical device. Consequently, FD-OCT systems are capable of higher data acquisition speeds. Axial depth measurement is achieved by Fourier transforming the spectra in one of two ways. The first approach is to use a broadband source and spectrometer. The interference pattern is dispersed immediately before detection; this process is called spectral domain SD-OCT. The main advantage of FD-OCT is the simultaneous measurement of all backscattering sites along the A-scan within the samples. To obtain an OCT image, the sample beam must be moved across the sample surface, with an A-scan acquired at each position of the beam. The set of A-scans is assembled to obtain an OCT image; this set of scans is called a B-scan. Accordingly, all depth information is acquired without the need for moving parts [21]. The acquired wavenumber-dependent data are converted into axial scan information by an inverse Fourier transform.

### 2.4. Femtosecond Laser Source—LDV Z8

The LDV Z8 operates on a mode-locked, diode-pumped, oscillator ytterbium-doped YAG laser (Yb:YAG) at a wavelength of 1030 nm, with crystals that have a bandwidth of 20–40 nm. Unique to the, LDV Z8 the cornea can be applanated, for example, in refractive corneal surgery or a liquid interface can be used, as in cataract surgery. The pulse energy is in the range of 50 nJ–25 μJ and has a pulse length of 200–350 fs, depending on the tissue treated. The system has an adaptive pulse management technology that permits an individual pulse energy, that is, more energy at the depth of the eye and less at the surface. An adaptive optical system is also available, which optimally focuses the laser spot in every plane.

Due to its large numerical aperture, the LDV Z8 can make small and very precise laser spots, though this requires a high pulse rate (in the MHz range). A smooth cut without tissue bridges is possible because the laser spots overlap. The tissue is processed by the laser from back to front. In the treatment algorithm, the lens fragmentation is performed first. This is possible because of the small spot size and small energies, which creates relatively small bubbles. In our study, the capsulotomy was done after lens fragmentation, followed by clear corneal incisions as the last step. This order has a great advantage, since bubbles do not absorb the laser energy before it is applied to the tissue. This approach delivers optimal cutting quality. Furthermore, the LDV Z8 is a fully mobile laser system, which can be easily used in various operating theaters, within the same vicinity and is operational within 30 min after being switched on [22,23].

### 2.5. Pre-, Intra-, Post-Operative Examinations and Surgical Methods

Intraoperative parameters such as surgical time (ST), phaco time, effective phaco time (EPT), vacuum time (VT) during femtosecond laser incisions, and the diameter of the capsulotomy/capsulorhexis including adverse effects were recorded. Upon occurrence, side effects were documented. All patients were implanted with a one-piece IOL NS-60YG (Nidek Co. Ltd., Gammagori, Aichi, Japan). The SRK/T formula was used to calculate the biometry.

Preoperatively, as well as, at 1 day, 12 days, 4 weeks, 8 weeks, and 12 weeks after the procedure, the best spectacle-corrected distant visual acuity (BSCVA) was measured with the spherical equivalent being calculated preoperatively, and 4 weeks and 8 weeks postoperatively.

#### 2.5.1. Femtosecond Laser Surgical Method

For the purpose of our research, we used the FEMTO LDV Z8 (Ziemer Ophthalmic Systems, Port, Switzerland) as the femtosecond laser platform. The eye was fixed with a target vacuum of 400 mbar with a suction ring. The handpiece of the laser was docked to the suction ring, whereby the eye and the laser head were connected by a liquid interface filled with balanced salt solution (BSS). The handpiece also contained a color Top View camera and an OCT system, which analyzed and imaged the ocular structures in real time. The treatment with femtosecond laser could be individually adapted to the patient’s eye. This possibility was enabled by the special role of imaging system, including the color Top View camera and an OCT system. The LDV Z8 femtosecond laser uses a spectral domain OCT. Both the Top View and the OCT images intraoperatively display the ocular structures precisely, as well as automatically place and visualize the planned resections, which could be fine-tuned during the procedure if deemed necessary. Furthermore, the Top View not only automatically recognizes the pupil and the limbus, but also all border structures, including blood vessels, pterygia, etc.

**Figure 2:**
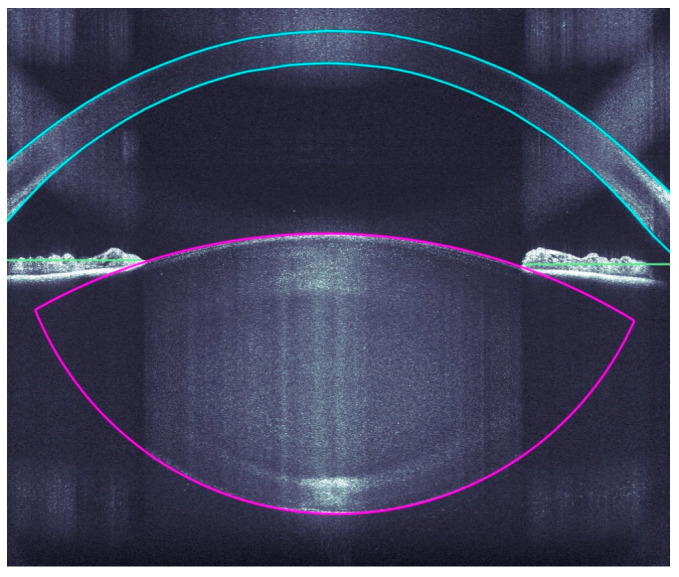
Intraoperative analyses of all ocular structure.

A very accurate OCT detection of the ocular structures allows a very precise incision of the clear corneal accesses, arcuate incisions, capsulotomies, and lens fragmentations by the Femtosecoond laser. The OCT imaging covers the corneal surfaces (anterior and posterior), iris, pupil, and anterior and posterior lens capsule (Figure 2).

**Figure 3:**
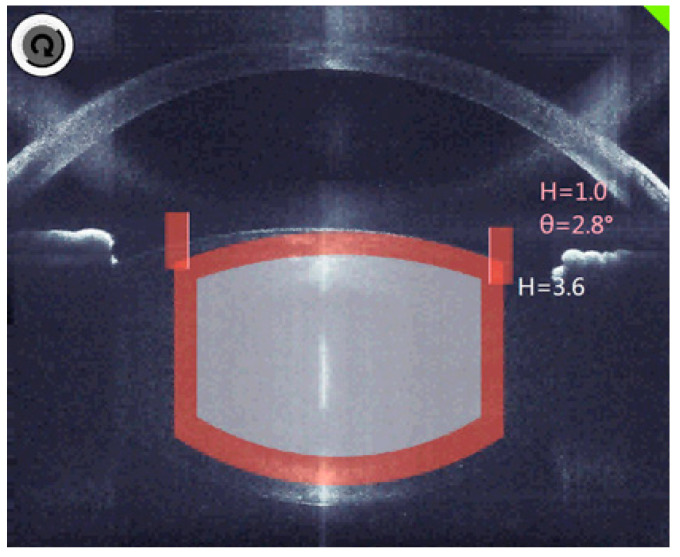
The planned cutting lines and areas are marked grey, the red bars are the calculated safety distances.

The decisive factor in OCT is the three-dimensional analysis, which fully identifies the anatomical structures and automatically suggests the cutting positions with the inclusion of an additional safety distance (Figure 3).

**Figure 4:**
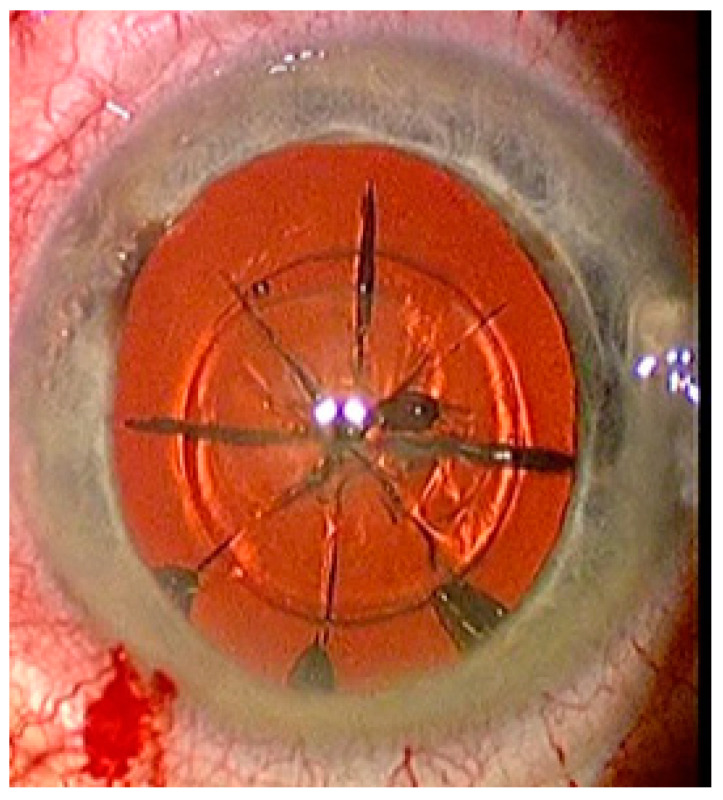
Status after femtosecond laser application with eight pie pieces lens fragmentation.

FEMTO LDV Z8 is a low-energy laser, which only generates a few bubbles and accordingly the treatment begins with lens fragmentation. A lens fragmentation pattern with eight pie pieces was set. Perpendicular to the pie pieces, a concentric circle could also be placed additionally (Figure 4). Following lens fragmentation, an anterior capsulotomy of 5 mm diameter is created.

A hydrodissection is then carried out to mobilize the cortical parts. With the phacoemulsification that follows, the pre-cut parts can be easily removed. With the irrigation suction, the cortex can be removed until a clear lens capsule is available. At the end of the surgery, an intraocular lens is implanted in the capsular bag.

#### 2.5.2. Conventional Phacoemulsification Method

A CataRhex 3 (Oertli Instrumente AG, Berneck, Switzerland) was used as a phacoemulsification device for the study. The device is equipped with an easyPhaco^®^ tip, which enables targeted emulsification through a focused axial release of ultrasound energy. In this surgical technique, a main incision of 2.2 mm was made with 2 additional paracenteses of 0.8 mm each. Viscoelastic (sodium hyaluronate 10 mg/mL) was used as a placeholder in the anterior chamber. With a cystotome, a continuous capsulorhexis was formed with a target diameter of 5 mm. After the hydrodissection was carried out, the cataract was removed using the stop and chop technique. The cortex remnants were aspirated using an irrigation/aspiration system. The IOL was then implanted into the capsular bag.

IBM SPSS Statistics Version 22.0 (IBM Corp., Armonk, NY, USA) was used for statistical analysis. Kolmogorov-Smirnov and Shapiro-Wilk tests were used to analyze the data sets to see if they were subject to a normal distribution. A data set was considered normally distributed if *p* > 0.05. The parametric data sets were further analyzed with Pearson normality test, unpaired *t*-test, RM one-way ANOVA, ordinary one-way ANOVA, D’Agostino, Pearson r-test, and the non-parametric datasets were further analyzed with the Friedmann test. Significance was achieved if *p* < 0.05.

## 3. Results

In the femtosecond laser group, 34 (52.3%) males and 31 (47.7%) females, and in the conventional group 27 (41.5%) male and 38 (58.5%) female patients were treated. The average age of patients in the femtosecond laser group was 70.5 ± 8.4 years (range 46–83) and 69.6 ± 8.0 years (range 43–85) in the conventional group.

**Figure 5:**
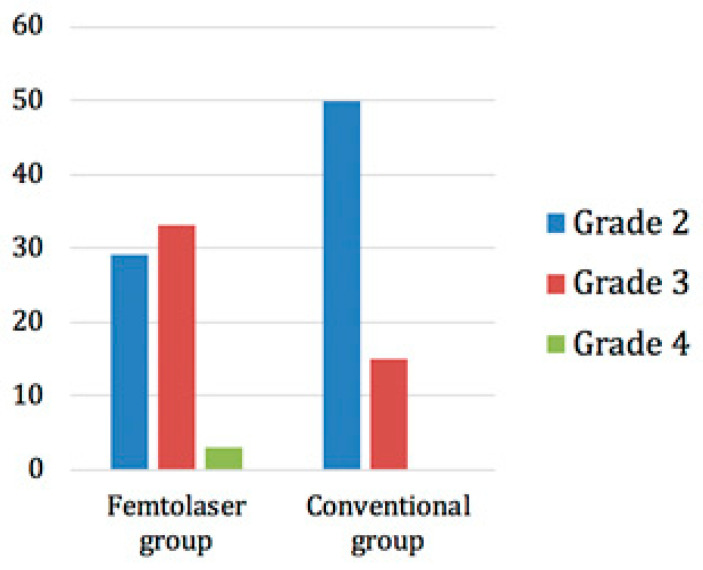
Distribution of cataract grades in the two groups.

The trial was randomized, which meant that patients were randomly allocated to one of the two treatment groups. The lens opacity grading was performed according to the Lens Opacities Classification System III (LOCS III). In the femtosecond laser group, 29 patients had a grade 2 cataract, 33 had a grade 3 cataract, and 3 had a grade 4 cataract. On the other hand, 50 patients in the conventional group had a grade 2 opacity and 15 had a grade 3 opacity (Figure 5).

Despite randomization, the subjects in this study were not stratified according to their preoperative cataract grade density. Of note is the fact, that (higher preoperative) cataract grade density (according to LOCS III grading system) was observed in the FLACS group. In the femtosecond laser group, the mean cataract density was grade 2.6 whilst in the conventional group it was grade 2.23. The difference was statistically significant (*p* < 0.001). The degree of cataract and the allocated surgical method showed a statistically significant association. (Pearson Chi-square: 15.332; df = 2; *p* < 0.001). The mean surgery time for femtosecond laser was 7.6 ± 1.2 min and for the conventional procedure 6.7 ± 1.7 min. The difference between the two methods was statistically significant (*p* = 0.004). The mean vacuum time for the femtosecond laser was 140 ± 26 s.

### 3.1. Corneal Parameter Analysis

#### 3.1.1. Endothelial Corneal Cell Density (CD)

The mean corneal endothelial cell count (CD) in the femtosecond laser group was 2440 ± 290 cells/mm^2^ (range 1580–2949) preoperatively, 2096 ± 513 cells/mm^2^ (range 691–2863) 4 weeks postoperatively, and 2130 ± 450 cells/mm^2^ (range 988–2791) 12 weeks postoperatively. In the conventional group, the mean CD preoperatively was 2379 ± 414 cells/mm^2^ (range 436–3084), after 4 weeks postoperatively, it was 1961 ± 517 cells/mm^2^ (range 797–2870), and after 12 weeks postoperatively, it was 1966 ± 567.7 cells/mm^2^ (range 603–2889) (Table 2).

At the preoperative starting point, there were no statistically significant differences between the study groups with regard to corneal endothelial cell count (*p* = 0.976). Additionally, at any point in time, no statistically significant difference between the femtosecond laser and conventional group in terms of corneal endothelial cell count was observed. However, in the femtosecond laser group, the corneal endothelial cell loss was meaningful, compared to the preoperative values 4 weeks postoperative (*p* = 0.0007) and 12 weeks postoperative (*p* = 0.0029). In the conventional group, postoperative corneal endothelial cell loss was highly significant with *p* < 0.0001, after 4 and 12 weeks.

The analysis of endothelial cell density was further split down by cataract grade. The mean (SD) endothelial cell density in patients with a preoperative cataract grade 2 decreased by 198.0 ± 236.5 in the femtosecond laser group and 381.3 ± 446.9 in the conventional group, at 4 weeks postoperatively. The difference between the femtosecond and conventional groups was statistically significant (*p* = 0.048). The difference in mean reduction in endothelial cell density from preoperative to 12 weeks postoperative between the two groups was, however, not statistically significant (*p* = 0.093) (Table 2).

The mean decrease in endothelial cell density from preoperative to 4 and 12 weeks postoperative in patients with a preoperative grade 3 cataract was more pronounced than in the preoperative grade 2 cataract patients, both in the femtosecond laser and conventional groups. In the femtosecond laser group, a reduction in endothelial cells of 435.6 ± 507.2 after 4 weeks and 390.2 ± 446.5 after 12 weeks was observed postoperatively. In the conventional group, the postoperative endothelial cell loss was 743.5 ± 640.6 after 4 weeks and 753.5 ± 742.0 after 12 weeks. However, no statistical difference was found between the groups at either 4 weeks (*p* = 0.097) nor 12 weeks (*p* = 0.052), in the cataract grade 3 analysis (Table 2).

In the pooled analysis of the preoperative cataract grade 2 and 3 patients, the femtosecond laser group showed a postoperative endothelial cell loss of 316.8 ± 410.0 at 4 weeks and 287.9 ± 359.1 at 12 weeks. In the conventional group, the postoperative endothelial cell loss was 461.8 ± 513.5 after 4 weeks and 430.9 ± 551.8 after 12 weeks. However, the difference did not any show statistical significance either at after 4 weeks (*p* = 0.094) nor at 12 weeks (*p* = 0.094) (Table 2).

The mean age of the patients participating in this study was 70.5 ± 8.3 years in the femtosecond laser group and 69.6 ± 8.1 years in the conventional group (*p* = 0.523). Split by cataract density grade 2, the mean age in the femtosecond laser group was 67.2 ± 8.2 years and in the conventional group it was 69.4 ± 8.5 years (*p* = 0.244). For grade 3, the mean age was 73.1 ± 7.5 years in the femtosecond laser group and 70.0 ± 6.7 years in the conventional group (*p* = 0.185). For the combined groups 2 and 3, the mean age was 70.2 ± 8.4 years in the femtosecond laser group and 69.6 ± 8.09 years in the conventional group (*p* = 0.666) (Table 2).

The mean anterior chamber depth (ACD) was 3.06 ± 0.39 mm in the femtosecond laser group and 3.03 ± 0.30 in the conventional group (*p* = 0.706). Broken down by cataract density grade, in the grade 2 cataract subjects, the ACD was 3.08 ± 0.40 in the femtosecond laser group and 2.98 ± 0.31 in the conventional group (*p* = 0.237). The mean ACD in grade 3 cataract subjects was 3.04 ± 0.37 in the femtosecond laser group and 3.20 ± 0.22 in the conventional group (*p* = 0.116). In the combined groups 2 and 3, the mean ACD was 3.06 ± 0.38 in the femtosecond laser group and 3.03 ± 0.30 in the conventional group (*p* = 0.724) (Table 2).

#### 3.1.2. The Coefficient of Variation of Endothelial Cell Area (CV)

The coefficient of variation of the endothelial cell area (CV) was 36.48 ± 4.86% for the femtosecond laser group, preoperatively, rising to 40.05 ± 5.24% 4 weeks, postoperatively, and to 38.90 ± 5. 28% 12 weeks, postoperatively. In the conventional group, the CV preoperatively, was 38.4 ± 6.61% and increased 4 weeks postoperatively to 41.46 ± 6.68%, and 12 weeks postoperatively to 38.57 ± 5.41%. Compared to the preoperative values, the CV 4 weeks postoperatively increased significantly in the femtosecond laser group (*p* = 0.008) and in the conventional group (*p* = 0.032), 4 weeks postoperatively; no statistical difference was found between the two groups (*p* = 0.934). On the other hand, the mean increase in the coefficient of variation of endothelial cell area at 12 weeks, postoperatively, was statistically significant between the groups (*p* = 0.034) (Table 3).

The statistical calculation of the subgroups analysis of the mean coefficient of variation of the endothelial cell area based on the analysis of the cataract grading yielded the following results.

The mean coefficient of variation of endothelial cell area in patients with grade 2 cataract increased in the femtosecond laser group preoperatively from 36.90 ± 4.41 to 40.07 ± 4.35 at 4 weeks postoperatively, and to 38.77 ± 3.87 at 12 weeks postoperatively. In the conventional group, the preoperative value was 39.25 ± 7.06 and increased to 40.61 ± 5.22 at 4 weeks postoperatively and to 39.06 ± 5.64 at 12 weeks postoperatively. There was no statically significant difference between the groups either at 4 weeks postoperatively (*p* = 0.218) or at 12 weeks postoperatively (*p* = 0.159) (Table 3).

The analysis of the patient group with cataract grade 3 showed the following results. In the femtosecond laser group, the mean (SD) coefficient of variation of endothelial cell area was 36.03 ± 5.50 preoperatively, increased to 40.14 ± 6.20 at 4 weeks postoperatively, and to 39.45 ± 6.40 at 12 weeks postoperatively. In the corresponding group of subjects treated with the conventional group of subjects, a value of 35.29 ± 3.17 was observed preoperatively, which increased to 44.43 ± 10.00 at 4 weeks postoperatively and to 36.86 ± 4.22 at 12 weeks. The difference between groups was statistically significant at 4 weeks (*p* = 0.20). However, the mean increases between the groups was not statistically significant at 12 weeks postoperatively (*p* = 0.203) (Table 3).

The mean increase in the coefficient of variation of cell area in the pooled analysis of grade 2 and 3 cataract patients was similar. In the femtosecond laser group, the preoperative value was 36.48 ± 4.95 and increased postoperatively to 40.11 ± 5.31 at 4 weeks and 39.10 ± 5.23 at 12 weeks. In the conventional group, the mean increase in the coefficient of variation of cell area was 38. 40 ± 6.61 and increased 4 weeks postoperatively to 41.46 ± 6.68 and 12 weeks postoperatively to 38.57 ± 5.41. No statistical difference was found between the groups, 4 weeks postoperatively (*p* = 0.976). However, at 12 weeks postoperatively, a statistical difference between the groups was observed (*p* = 0.018) (Table 3).

#### 3.1.3. The Percentage of Hexagonal Cells (6A)

The mean value of the percentage of hexagonal cells (6A) in the femtosecond laser group was 47.11 ± 7.01% preoperatively, 41.07 ± 6.27% 4 weeks postoperatively, and 43.06 ± 6.58% 12 weeks postoperatively. In the conventional group, the 6A value 46.31 ± 8.62% was observed, 4 weeks postoperatively 39.03 ± 7.15%, and 12 weeks postoperatively 41.46 ± 8.07%. In the femtosecond laser group, a significant reduction was observed 4 weeks postoperatively (*p* < 0.001) and 12 weeks postoperatively (*p* = 0.0262), compared to preoperative 6A values. In the conventional group, the 6A values also decreased in a significant manner after 4 weeks postoperatively (*p* < 0.001) and 12 weeks postoperatively (*p* = 0.003). However, it was found that there was no statistical difference in the mean value of the percentage of hexagonal cells (6A) between the groups at 4 weeks (*p* = 0.262) and 12 weeks (*p* = 0.449), postoperatively.

Further analysis of the mean decrease in the percentage of hexagonal cells (6A) was performed according to the cataract grade.

In patients with cataract grade 2, there was a mean decrease in the percentage of hexagonal cells (6A) in the femtosecond laser group from 46.23 ± 6.21 preoperatively to 42.07 ± 6.33 at 4 weeks postoperatively, and to 43.67 ± 6.33 at 12 weeks postoperatively. In the conventional group, the preoperative value was 45.41 ± 8.85 and decreased to 39.45 ± 7.44 by postoperative week 4, and to 41.10 ± 7.96 by postoperative week 12. There was no statistical difference between the groups either at 4 weeks postoperative (*p* = 0.191) nor at 12 weeks postoperative (*p* = 0.249) (Table 4).

Patients with cataract grade 3 showed a similar trend. In the femtosecond laser group, the percentage of hexagonal cells (6A) was 48.23 ± 7.98 preoperatively and decreased to 39.89 ± 6.40 at 4 weeks postoperatively and to 42.55 ± 6.81 at 12 weeks. In the conventional group, the preoperative value was 49.57 ± 7.07 and decreased to 37.57 ± 6.03 at 4 weeks postoperatively and to 42.71 ± 8.63 at 12 weeks; it was 57 ± 7.07 and decreased to 37.57 ± 6.03 at 4 weeks postoperatively and to 42.71 ± 8.63 at 12 weeks. No statistical difference was found between the groups at 4 weeks postoperatively (*p* = 0.214) and 12 weeks postoperatively (*p* = 0.648) (Table 4).

In the pooled group analysis of patients with cataract grade 2 and 3, the preoperative value in the femtosecond laser group was 47.21 ± 7.15 and decreased to 40.98 ± 6.40 at 4 weeks postoperatively and to 43.12 ± 6.54 at 12 weeks postoperatively. The baseline value in the conventional group was 46.31 ± 8.62 and decreased to 39.03 ± 7.15 at 4 weeks postoperatively, and to 41 at 12 weeks postoperatively. The mean decrease in the percentage of hexagonal cells from preoperative to 4 and 12 weeks postoperative was numerically higher in the conventional technique group than in the femtosecond laser group, but no statistical difference between the groups could be detected either during 4 weeks postoperative (*p* = 0.325) or after 12 weeks postoperative treatment (*p* = 0.473) (Table 4).

### 3.2. The Effective Phacoemulsification Time (EPT)

The effective phacotime (EPT) was 1.52 ± 1.83 s for the femtosecond laser group (range 0.08–8.8) and 1.76 ± 1.90 s for the conventional group (range 0.24–11.20). Regarding EPT, no significant differences were found (*p* = 0.093) between groups. The correlation analysis showed the existence of a linear relationship between EPT and CD 4 weeks postoperatively (r = −33.3%, *p* < 0.001) and 12 weeks postoperatively (r = −33.7%, *p* < 0.001) in both groups.

A further analysis of EPT as per cataract grade was performed. In patients with a cataract grade 2, the EPT was 0.98 ± 1.19 in the femtosecond laser group and 1.44 ± 1.17 in the conventional group. The EPT was statistically significantly shorter in the femtosecond laser group, as compared to the conventional group (*p* = 0.007).

In patients with cataract grade 3, an EPT of 1.79 ± 2.02 was observed in the femtosecond laser group and an EPT of 2.92 ± 3.27 in the conventional group. Although the mean EPT was shorter in the femtosecond laser group than in the conventional group, no statistical difference was observed between the groups (*p* = 0.364).

In the pooled analysis of patients with cataract grade 2 and 3, the mean EPT was 1.38 ± 1.69 in the femtosecond laser group and 1.76 ± 1.91 in the conventional group. The EPT was significantly shorter in the femtosecond laser group than in the conventional group (*p* = 0.034) (Table 5).

There were only 3 cataract grade 4 patients, all in the FLACS group.

### 3.3. Visual Acuity and Refraction

**Figure 6:**
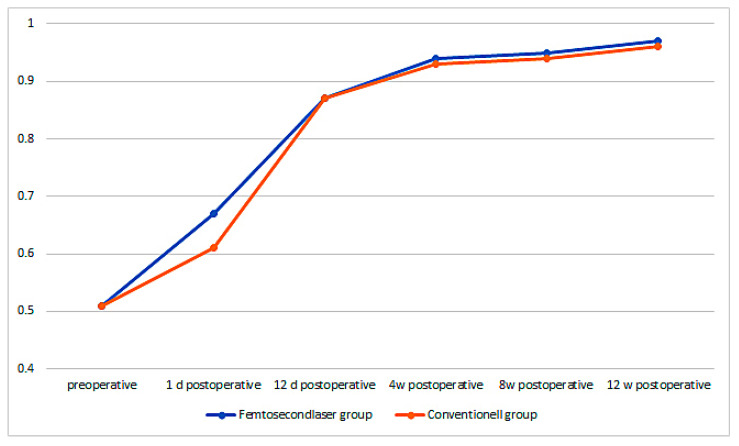
Best corrected visual acuity (BCVA) over time for both groups.

The preoperative best corrected visual acuity (BCVA) in the femtosecond laser group was 0.51 ± 0.15 and increased on the first postoperative day to 0.67 ± 0.31, 12 days after it increased to 0.87 ± 0.17, then after 4 weeks it increased to 0.94 ± 0.14, 8 weeks postoperative to 0.95 ± 0.10, and finally 12 weeks postoperative, it increased to 0.97 ± 0.12. The increase in BCVA from preoperative to 1 day postoperative was already highly significant (*p* < 0.001). In the conventional group, the BCVA was 0.51 ± 0.15 and increased 1 day postoperatively to 0.61 ± 0.29, 12 days postoperatively to 0.87 ± 0.20, 4 weeks postoperatively to 0.93 ± 0.15, 8 weeks postoperatively to 0.94 ± 0.15, and 12 weeks postoperatively to 0.96 ± 0.14. There was a significant difference in the first postoperative day, the BCVA increased significantly higher in the femtosecond laser group than in the conventional group (*p* = 0.038). However, during later time-points of the follow-up, no significant difference between the groups could be observed anymore (Figure 6).

The mean absolute error (MAE) is defined as the difference between the predicted and achieved postoperative spherical equivalence refraction. In the femtosecond laser group, the MAE was −0.20 ± 0.49 D (range −1.46–1.11) and in the conventional group it was −0.06 ± 0.52 D (range −1.23–0.98). There was no statistically significant difference between the surgical methods (*p* = 0.121), with regard to the observed MAE values.

### 3.4. Capsulotomy/Capsulorhexis

The target diameter for capsulotomy as well as capsulorhexis was set at 5 mm. The mean capsulotomy diameter for the femtosecond laser group was 5.0 ± 0.1 mm (range 4.8–5.4 mm) and capsulorhexis for the conventional group was 4.7 ± 0.4 mm (range 4.0–5.6 mm). The femtosecond laser capsulotomy was statistically significantly more precisely than the manual capsulorhexis procedure (*p* < 0.0001, t = 5.734, df = 127).

## 4. Discussion

In our scientific study, we used a modern, low-energy, femtosecond laser that could be applied very precisely and gently due to its small spots, not least because of the high-resolution spectral-domain OCT, which in fact serves as a navigation system during the surgery and incision process. Within the scope of the study, we used one of the latest generation of femtosecond lasers, with the aim of demonstrating that the subtle and gentle surgical method has advantages over a conventional approach in the context of a clinical application. In our study, we compared the clinical outcomes between femtosecond laser assisted cataract surgery (FLACS) and conventional phacoemulsification, focused on corneal endothelial cell behavior. We found that despite highly significant denser lens opacity in the femtosecond laser group, 2.60 ± 0.58 versus conventional group 2.23 ± 0.42 (*p* < 0.001), the corneal endothelial cell count did not show a statistically significant difference between the treated groups at any time. In the femtosecond laser group, corneal endothelial cell loss after 12 weeks was 12.7% and in the conventional group it was 17.4%. The effective phacotime (EPT) in the femtosecond laser group was 1.62 ± 1.85 s lower than in the conventional group with 1.76 ± 1.90 s, but the difference was not statistically significant. In the analysis of the subgroups, differences in the EPT could nevertheless be observed. The EPT was statistically significantly shorter in the FLACS group in cataract grade 2 patients, as compared to the conventional technique group. The EPT was also shorter in the FLACS group (compared to the conventional technique group) in cataract grade 3 patients, although not statistically significant. There were 3 cataract grade 4 patients, all in the FLACS group. Our study also provides some evidence that FLACS has a positive effect on corneal endothelial cell preservation, as despite the preoperative denser lens opacity in the femtosecond laser group, no differences in postoperative endothelial cell counts, coefficient of variation of endothelial cell area and percentage of hexagonal cells were found between the two groups. This was further corroborated by the outcomes of the subgroup analysis, based on the cataract grade of the endothelial cell behavior. Especially in grade 2 cataract, the largest subgroup, there was a consistent correlation between a smaller EPT in the FLACS group and reduced corneal endothelial cell loss. In addition, our subgroup analysis of the coefficient of variation of the endothelial cell area (CV) corroborated the relationship between EPT and endothelial cell loss, i.e., the lesser the EPT, the smaller the change coefficient of variation of the endothelial cell area (CV). Further positive effects were that the best corrected visual acuity (BCVA) recovered significantly faster in the femtosecond laser group at least on the first postoperative day, and that capsulotomy was significantly more precise in the FLACS group, as compared to the conventional capsulorhexis.

The significantly better improvement in visual acuity from pre-op to one-day post-operation in the FLACS group might reveal that the visual recovery and rehabilitation time in the first postoperative day was faster. With today’s fast paced lifestyle demands; patients are eager to return to their workplaces and everyday activities as soon as possible.

Published research results described numerous advantages of femtosecond laser-assisted cataract surgery over conventional methods. Nevertheless, at least today, conventional cataract surgery is still the golden standard medical practice, and FLACS is approaching broad scientific acceptance. The surgical result of conventional cataract surgery using phacoemulsification has a flat learning curve and depends on the skills and experience of the surgeons, which is presumably not the case with the use of femtosecond lasers in cataract surgery, where a much steeper learning curve even for less experienced surgeons is usually observed [24]. Another study showed that inexperienced surgeons use significantly more time and ultrasound energy in conventional cataract surgery, as compared to experienced surgeons [25]. However, it should be noted that an experienced surgeon learns FLACS much faster than a less experienced one.

It was long generally accepted that ultrasound energy and fluid dynamic flow have a negative effect on eye structures. The damage is caused by intraocular manipulations, the ultrasound energy is converted into heat and the circulating BSS fluid in the anterior chamber [26]. There is also a direct link between EPT and corneal endothelial cell loss. Corneal endothelial cell loss is reported in the literature to be between 1.4% and 23% [27,28,29,30,31], which can be further supported by our results. The viscoelastic potentially plays a protective role of the corneal endothelial cells [32]. This might be an important factor in addition to EPT, total surgery time and intraocular manipulation, which is why such a wide range of corneal endothelial cell loss is identified in the literature. In our study, the variable related to the use of a viscoelastic was mitigated by the use of the same product in both study groups.

As mentioned above, the use of the femtosecond laser in our study had a significant advantage in terms of the precision achieved for capsulotomy. This might influence the clinical outcome. Optimal IOL centration could have a positive effect on the prevention of postoperative aberration. It was shown that accurate, symmetrical capsulorhexis is causally related to effective lens positioning. A difference of only 1 mm in IOL positioning could result in a change in refraction of about 1.25 dioptres [33].

In the same study, it was shown that after 1–3 days postoperatively, there was no significant difference in corneal endothelial cell loss between femtosecond laser with 249 cells/mm^2^ (SD ± 744) (9.1%) and the conventional groups with 235 cells/mm^2^ (SD ± 681) (8.2%) (*p* = 0.87). The same was observed after three months postoperative FLACS versus conventional procedure with 274 cells/mm^2^ (SD ± 358) (11.4%) versus 333 cells/mm^2^ (SD ± 422) (13.9%) (*p* = 0.30), respectively. The association between EPT and corneal endothelial cell loss was also investigated. The correlation between corneal endothelial cell loss and phacoenergy was found to be highly significant. A linear dependence between EPT and corneal endothelial cell loss was described previously [33]. This trend was also further supported in a recent study where corneal endothelial cell loss was statistically significantly lower in the FLACS group, as compared to the conventional group, i.e., 288 ± 424 cells/mm^2^ versus 443 ± 356 cells/mm^2^, at 18 months follow-up (*p* = 0.017) [34]. Other studies showed that in corneal endothelial cell loss, not only the cumulative ultrasound energy but also the irrigation/aspiration time and the amount of BSS consumed are important [35,36]. We found that corneal endothelial cell loss was multifactorial. However, FLACS might have the advantage of reducing intraocular manipulation during surgery in cases of preformed accesses, capsulotomy, and lens fragmentation [37,38,39].

In one of the recent publications with a meta-analysis of a total of 73 studies comparing the FLACS and CUCS, both methods showed efficacy and safety. Although, at the same time in eyes treated with FLACS, uncorrected and corrected distance visual acuities and spherical equivalence after 1 month to 3 months (*p* = 0.04, *p* = 0.005, and *p* = 0.007, respectively) were better, total and effective phacoemulsification times were shorter (*p* < 0.001 each), cumulative dissipated energy was less (*p* < 0.001), circularity was more accurate (*p* < 0.001), central corneal thickness after 1 day and 1 month to 3 months was less (*p* < 0.001 and *p* = 0.004, respectively), and endothelial cell loss after 3 to 6 weeks and 3 months was less (*p* = 0.002 and *p* < 0.001, respectively), as compared to CUCS [40].

A recent clinical trial demonstrated that postoperative CD between groups (2211.88 ± 392.49 CUCS; 2246.31 ± 403.48 FLACS) was not statistically significant (*p* = 0.869). The reason might be that a low-frequency femtosecond laser was used with a pulse energy in the µJ range and that a longer application time was necessary due to lens fragmentation into small parts by the femtosecond laser and thus more energy was transmitted into the tissue. Total ultrasound time, torsional energy time, cumulative dissipated energy, and fluid requirements were significantly lower in the FLACS group (*p* < 0.05). Considering that the other parameters of FLACS were significantly lower as compared to CUCS, a further development of the fragmentation strategy would need to be optimized with a consecutive smaller total pulse energy application [41]. In another paper, the problem of incomplete removal of the lens fragments were shown, which could consecutively lead to chronic inflammation and corneal edema and the reduction of CD. It is mainly the small lens fragments that are not visible after surgery and can only be detected later [42]. Such clinical experience should also be incorporated into the optimization of lens fragmentation procedure when using femtosecond lasers. These further increase the application safety.

In any case, the introduction of the femtosecond laser as a surgical tool in ophthalmology paves the way towards further refining surgical methods. A particularly important aspect was that the imaging techniques such as intraoperative OCT and its analysis keep pace with the effective surgical femtosecond laser to achieve the most effective clinical outcome.

## 5. Conclusions

Low energy FLACS performed better with regards to endothelial cell loss, endothelial cell size and shape variations, and effective phaco time in patients with certain cataract grade. Due to higher cataract grade in the FLACS group, entire sample analysis did not reveal differences between the two groups. FLACS had a positive effect on endothelial cells preservation and was significantly more precise, particularly creating accurately sized capsulotomy. FLACS shows many advantages in clinical use compared to manual cataract surgery, especially in the precision and protection of the tissue. These advantages lie in various areas of implementation, such as the formation of clear corneal incisions, arcuate incisions, capsulotomy, and the reduction of the applied ultrasonic energy through prior lens fragmentation. Since its introduction, the femtosecond laser technology has gained exciting new perspectives.

## Figures and Tables

**Table 1:** Terms and abbreviations mentioned more than once in the manuscript.

ACD	Anterior Chamber Depth
BSCVA	Best spectacle-corrected distant visual acuity
BSS	Balanced salt solution
CCT	Central corneal thickness
CD	Density of endothelial cells per mm^2^
CUCS	Conventional ultrasound cataract surgery
CV	Coefficient of variation of endothelial cell area
ECC	Endothelial cell count
EPT	Effective phaco time
FD	Fourier domain
FLACS	Femtosecond laser-assisted cataract surgery
IOL	Intra-Ocular Lens
LOCS III	Lens Opacities Classification System III
NUM	Absolute number of endothelial cells
OCT	Optical coherence tomography
SD	Spectral domain
SRK/T formula	Retzlaff JA, Sanders DR, and Kraff MC/Theoretical formula representing a combination of linear regression method with a theoretical eye model
ST	Surgical time

**Table 2:** Comparison of corneal endothelial cell density (CD, in cells/mm^2^), the age of subjects, and the ACD between the femtosecond laser and the conventional group, over time for all eyes, and split into subgroups.

	Femtosecond Laser Group	Conventional Group	*p*-Value
**All grades**			
Pre-operative	2440 ± 290.2	2379 ± 413.7	*p* = 0.976
4 weeks postop	2096 ± 513.3	1961 ± 516.5	*p* = 0.166
12 weeks postop	2130 ± 450.3	1966 ± 567.7	*p* = 0.177
Age (years)	70.5 ± 8.3	69.6 ± 8.1	*p* = 0.523
ACD (mm)	3.06 ± 0.39	3.03 ± 0.30	*p* = 0.706
**Grade 2**			
Pre-operative	2469 ± 315	2332 ± 445	
4 weeks postop	2277 ± 417	2006 ± 499	*p* = 0.048
12 weeks postop	2289 ± 371	2015 ± 528	*p* = 0.093
Age (years)	67.2 ± 8.2	69.4 ± 8.5	*p* = 0.244
ACD (mm)	3.08 ± 0.40	2.98 ± 0.31	*p* = 0.237
**Grade 3**			
Pre-operative	2431 ± 266	2548 ± 205	
4 weeks postop	1988 ± 529	1804 ± 566	*p* = 0.097
12 weeks postop	2045 ± 443	1794 ± 694	*p* = 0.052
Age (years)	73.1 ± 7.5	70.0 ± 6.7	*p* = 0.185
ACD (mm)	3.04 ± 0.37	3.20 ± 0.22	*p* = 0.116
**Grades 2 and 3**			
Pre-operative	2450 ± 290	2378 ± 414	
4 weeks postop	2132 ± 494	1961 ± 517	*p* = 0.094
12 weeks postop	2169 ± 423	1965 ± 568	*p* = 0.094
Age (years)	70.2 ± 8.4	69.6 ± 8.09	*p* = 0.666
ACD (mm)	3.06 ± 0.38	3.03 ± 0.30	*p* = 0.724

**Table 3:** Comparison of the coefficient of variation of the endothelial cell area (CV) in % between the femtosecond laser and conventional group over time, for all eyes and split into subgroups.

	Femtosecond Laser Group	Conventional Group	*p*-Value
**All grades**			
Pre-operative	36.48 ± 4.86	38.40 ± 6.61	
4 weeks postop	40.05 ± 5.24	41.46 ± 6.68	*p* = 0.934
12 weeks postop	38.90 ± 5.28	38.57 ± 5.41	*p* = 0.034
**Grade 2**			
Pre-operative	36.90 ± 4.41	39.25 ± 7.06	
4 weeks postop	40.07 ± 4.35	40.61 ± 5.22	*p* = 0.218
12 weeks postop	38.77 ± 3.87	39.06 ± 5.64	*p* = 0.159
**Grade 3**			
Pre-operative	36.03 ± 5.50	35.29 ± 3.17	
4 weeks postop	40.14 ± 6.20	44.43 ± 10.00	*p* = 0.020
12 weeks postop	39.45 ± 6.40	36.86 ± 4.22	*p* = 0.203
**Grades 2 and 3**			
Pre-operative	36.48 ± 4.95	38.40 ± 6.61	
4 weeks postop	40.11 ± 5.31	41.46 ± 6.68	*p* = 0.976
12 weeks postop	39.10 ± 5.23	38.57 ± 5.41	*p* = 0.018

**Table 4:** Comparison of the mean value of the percentage of hexagonal cells (6A) in % between the femtosecond laser and conventional group over time, for all eyes and split into subgroups.

	Femtosecond Laser Group	Conventional Group	*p*-Value
**All grades**			
Pre-operative	47.11 ± 7.01	46.31 ± 8.62	
4 weeks postop	41.07 ± 6.27	39.03 ± 7.15	*p* = 0.262
12 weeks postop	43.06 ± 6.58	41.46 ± 8.07	*p* = 0.449
**Grade 2**			
Pre-operative	46.23 ± 6.21	45.41 ± 8.85	
4 weeks postop	42.07 ± 6.33	39.45 ± 7.44	*p* = 0.191
12 weeks postop	43.67 ± 6.33	41.10 ± 7.96	*p* = 0.249
**Grade 3**			
Pre-operative	48.23 ± 7.98	49.57 ± 7.07	
4 weeks postop	39.89 ± 6.40	37.57 ± 6.03	*p* = 0.214
12 weeks postop	42.55 ± 6.81	42.71 ± 8.63	*p* = 0.648
**Grades 2 and 3**			
Pre-operative	47.21 ± 7.15	46.31 ± 8.62	
4 weeks postop	40.98 ± 6.40	39.03 ± 7.15	*p* = 0.325
12 weeks postop	43.12 ± 6.54	41.46 ± 8.07	*p* = 0.4728

**Table 5:** EPT in seconds by cataract grade subgroups.

	Femtosecond Laser Group	Conventional Group	*p*-Value
**All grades**	1.52 ± 1.83	1.76 ± 1.90	*p* = 0.093
**Grade 2**	0.98 ± 1.19	1.44 ± 1.17	*p* = 0.007
**Grade 3**	1.79 ± 2.02	2.92 ± 3.27	*p* = 0.364
**Grades 2 and 3**	1.38 ± 1.69	1.76 ± 1.90	*p* = 0.035

## Data Availability

The data presented in this study are available on request from the authors, in particular the datasets are archived in the clinics treated. The data are not publicly available as they contain information that could compromise the privacy of the participants.
